# Primordial Prevention and Wearable Health Devices: The Wearables in Cardiology

**DOI:** 10.5935/abc.20160094

**Published:** 2016-06

**Authors:** Eduardo Campos Pellanda, Lucia Campos Pellanda

**Affiliations:** 1 Pontifícia Universidade Católica-Rio Grande do Sul - PUC-RS; Porto Alegre, RS - Brazil; 2 Instituto de Cardiologia/ Fundação Universitária de Cardiologia do Rio Grande do Sul - IC-FUC/RS; Porto Alegre, RS - Brazil; 3 Universidade Federal de Ciências da Saúde de Porto Alegre - UFCSPA, Porto Alegre, RS - Brazil

Epidemiology has shown the growing importance of prevention as a way of dealing with the cardiovascular risk epidemic. One of the fundamental changes in concepts in recent decades was the shift in the prevention paradigm to ever earlier stages, both in the disease development, affecting an increasing number of individuals that have yet to develop a pathological condition or those who are in the early and treatable stages, as of the life line itself, looking for the origin of chronic diseases in intrauterine life.^1^ Thus, the concept of primordial prevention arose, that is, the prevention of the actual risk factors.

Ideally, preventing the establishment of risk factors in a population would keep it disease-free. Although utopian, the scenario described is useful to guide prevention strategies. One of the main current objectives is to reach the greatest possible number of individuals before they develop a risk profile.

In this sense, the revolution of wearables, or wearable devices, has great potential to contribute to primary prevention, by participating in the daily lives of individuals with an all-inclusiveness that is impossible for isolated interventions based solely on health services to achieve.

To understand the wearables, one must recall how the computer ceased to be something detached to become something that is always close to our bodies. The computational device formats have evolved since the late 1970s, with the introduction of the concept of personal machines, or personal computers - the PCs. The term "personal" refers to a computing device attached to the daily lives of individuals, not corporate life. If they conquered the homes in the 1980s, in the middle of the 1990s, there were devices of several sizes and formats powered by batteries that allowed user mobility that was unheard of. While wireless networks (data or voice) expanded their reach, the telephone started to be carried along by people and even steal the attention that was previously given to the radio and watch.

This infrastructure allowed a radical change to occur in the interface of the common user and computing, also resulting in the birth of a mobile application ecosystem in this new form of Internet access - the main one, for many individuals. This mobile connection status is "always-on" and hyperpersonal,^2^ in addition to allowing new forms of communication in the metropolis environment. The logical evolution would be the intense use of mobile communication, as well as the miniaturization of components and devices, which are used attached to the body and not in pockets anymore.^3,4^

Thus, wearables can be not only always close to the individual, but also collect, through sensors, the body's own information, such as counting steps or measuring heart rate. Because of the very small screens, or without displays, they are at the same time, the smartphone extensions and information entry points through voice.^5^

At this initial phase, they take familiar forms, such as glasses (Google Glass) or watches (Apple Watch and Pebble, among others). In both cases, there is an adaptation of the original shapes of glasses and watches to process information. Watches would potentially have lower resistance, as they always provided some kind of extra information in addition to the hour. As for glasses, they were always perceived as intended for the amplification and correction of optical realities, which makes their transformation less acceptable socially at this historical moment.

At the moment, what seems clear is that people have different points of information in their bodies, thus making the perception of connection more natural and "invisible".

The worldwide sales of wearables should reach the figure of 200 million units by 2019.^6^ Considering today's scenario, we must think of wearables not only as a wearable accessory, but as an information accessory that can help the public or by capturing or reporting data. These two possibilities and their combinations allow more than just uploading data at times when people are not in front of the screens, in addition to the new useful characteristic that information on the wrist, for instance, can carry.

Simple data capture devices, such as straps with motion and heart rate sensors, can generate a huge volume of information, which allows not only individual patient monitoring, but also the planning of collective prevention strategies. This type of Big Data can be useful, for instance, to evaluate ecological interventions, such as changing the urban layout, to promote physical activity in a city's population.^7^

Additionally, the monitoring itself can help change behaviors, as it furthers individuals' knowledge about themselves and encourages them to take the leading role when caring for their own health.^8^

This is another example of the need for new paradigms in research on prevention. To be able to follow the evolution of concepts in prevention and the change in lifestyles at a time of mobile information, research also needs to be innovative, transdisciplinary and agile. The Brazilian Archives of Cardiology, in its role of stimulating innovative research in cardiology, proposes to carry out intervention studies in our country, so that technological innovations can in fact contribute to the improvement of health outcomes.
